# Enhancing the Evidence with Algorithms: How Artificial Intelligence Is Transforming Forensic Medicine

**DOI:** 10.3390/diagnostics13182992

**Published:** 2023-09-19

**Authors:** Alin-Ionut Piraianu, Ana Fulga, Carmina Liana Musat, Oana-Roxana Ciobotaru, Diana Gina Poalelungi, Elena Stamate, Octavian Ciobotaru, Iuliu Fulga

**Affiliations:** Faculty of Medicine and Pharmacy, Dunarea de Jos University of Galati, 35 AI Cuza St., 800010 Galati, Romania; alin.piraianu@gmail.com (A.-I.P.); carmina.musat@ugal.ro (C.L.M.); roxana_hag@yahoo.com (O.-R.C.); dianapoalelungi10@gmail.com (D.G.P.); coctavian72@gmail.com (O.C.); fulgaiuliu@yahoo.com (I.F.)

**Keywords:** artificial intelligence, medicine, forensic science, forensic medicine, pathology

## Abstract

Background: The integration of artificial intelligence (AI) into various fields has ushered in a new era of multidisciplinary progress. Defined as the ability of a system to interpret external data, learn from it, and adapt to specific tasks, AI is poised to revolutionize the world. In forensic medicine and pathology, algorithms play a crucial role in data analysis, pattern recognition, anomaly identification, and decision making. This review explores the diverse applications of AI in forensic medicine, encompassing fields such as forensic identification, ballistics, traumatic injuries, postmortem interval estimation, forensic toxicology, and more. Results: A thorough review of 113 articles revealed a subset of 32 papers directly relevant to the research, covering a wide range of applications. These included forensic identification, ballistics and additional factors of shooting, traumatic injuries, post-mortem interval estimation, forensic toxicology, sexual assaults/rape, crime scene reconstruction, virtual autopsy, and medical act quality evaluation. The studies demonstrated the feasibility and advantages of employing AI technology in various facets of forensic medicine and pathology. Conclusions: The integration of AI in forensic medicine and pathology offers promising prospects for improving accuracy and efficiency in medico-legal practices. From forensic identification to post-mortem interval estimation, AI algorithms have shown the potential to reduce human subjectivity, mitigate errors, and provide cost-effective solutions. While challenges surrounding ethical considerations, data security, and algorithmic correctness persist, continued research and technological advancements hold the key to realizing the full potential of AI in forensic applications. As the field of AI continues to evolve, it is poised to play an increasingly pivotal role in the future of forensic medicine and pathology.

## 1. Introduction

The next revolution from an IT and industrial point of view will be the use of artificial intelligence for multidisciplinary progress. Kaplan and Haenlein define AI as “the ability of a system to correctly interpret external data, learn from such data, and use it to achieve specific goals and tasks through flexible adaptation” [1]. The term “artificial intelligence” is used colloquially to describe machines that mimic the “cognitive” functions that humans associate with other human minds, such as “learning” and “problem solving” [2]. We are currently at the point of implementation of the fourth industrial revolution, with AI becoming a cornerstone for all digital transformation initiatives [3]. 

Algorithms are well-defined sets of instructions or steps that a computer follows to solve a particular problem or perform a particular task. These instructions are defined logically and sequentially so that they can be followed by the computer to achieve a particular result. In the context of the use of artificial intelligence in forensics, algorithms can be programmed to analyze data, recognize patterns, identify anomalies, or provide suggestions and decisions based on the data provided. These sets of instructions and mathematical operations can be used by artificial intelligence to perform specific tasks within the field of forensics.

In medicine, AI is already used as an assistant to doctors, to establish a correct diagnosis, detect and monitor vital signs, and even detect skin cancer [4]. Forensic medicine is a medical discipline that aims to prepare scientific medical-biological evidence for the application of judicial rules. Medical science, implicitly forensic medicine and pathology, cannot ignore the new expertise techniques. The classic way of performing an autopsy and drawing up an expert report has many limitations, but these can be reduced with the help of artificial intelligence. Forensic medicine and pathology changes in “the big data era”, and the development is a full expansion in 2023 artificial intelligence, bringing new opportunities. In recent years, numerous studies based on artificial intelligence technology have been carried out, such as face recognition, age and sex estimates, DNA analysis, postmortem interval estimation, and injury and cause of death identification, demonstrating the feasibility and advantages of using artificial intelligence technology in forensic medicine and pathology [5]. Thus, a new worldwide direction has been launched, which includes technology adaptability challenges with the potential of integration into the well-known medico-legal practice until now.

### 1.1. Definitions of Terms Related to AI and Medicine

#### 1.1.1. Artificial Intelligence

Alan Turing, the founding father of artificial intelligence gave the first definition of artificial intelligence as the science and engineering of making intelligent machines, especially intelligent computer programs [6]. According to Salto-Tellez M. et al. [7], AI is an advanced technological range of machines that can extract meaning and understanding from extended data inputs in ways that mimic human capabilities. Currently, a specific definition can be the ability of a system to interpret external data correctly and to learn from it to achieve specific goals and tasks through flexible adaptation [1].

#### 1.1.2. Machine Learning (ML)

Machine learning is a statistical technique of fitting models to data and, learning by training models using data [8]. In medicine, the most widely used application of machine learning is precision medicine (predicting the treatment that is likely to have the best effect on the patient) [9]. For precision medicine to work, a set of training data is required for which the outcome variable (e.g., disease onset) is known, which is called supervised learning.

#### 1.1.3. Deep Learning (DL)

Deep Learning, a subcategory of machine learning, is a deep neural network that has a specific configuration in which neurons are organized in several successive layers, which can independently learn representations of the data and progressively extract complex features to perform tasks such as computer vision and natural language processing (NPL) and is used in medicine to detect diseases from medical imaging [10].

#### 1.1.4. Natural Language Processing (NPL)

Natural language processing is a theoretically motivated range of computational techniques for analyzing and representing naturally occurring texts at one or more levels of linguistic analysis to achieve human-like language processing for a range of tasks or applications, being used in medicine to structure information in healthcare systems and extract relevant information from narrative texts to provide data for decision making [11].

#### 1.1.5. Robotics 

The robot has been defined as “a reprogrammable multifunctional manipulator designed to move material, parts, tools, or specialized devices through variable programmed motions for the performance of a variety of tasks” by the Robot Institute of America; in medicine, they are used for their precision, especially in surgical specialties [12].

#### 1.1.6. Artificial Neural Network (ANN)

Artificial neural networks are a class of artificial intelligence algorithms that emerged in the 1980s as a result of developments in cognitive and computer science research. Like other artificial intelligence algorithms, ANNs have been motivated to address different aspects or elements of learning, such as learning mode, induction mode, and inference mode [13].

#### 1.1.7. Convolutional Neural Networks (CNNs)

Convolutional neural networks are neural networks similar to regular neural networks because they are also composed of neurons with learnable weights. CNNs make the explicit assumption that inputs have specific structures like images. This allows for encoding of this property into the architecture by sharing the weights for each location in the image and having neurons respond only locally [14].

## 2. Literature Review

### 2.1. Methodology

We conducted a review of current literature including original articles and reviews that studied various clinical applications of AI in forensic medicine and pathology. We performed extensive searches on Google Scholar, PubMed, and ScienceDirect databases to identify relevant manuscripts. As keywords, we used “artificial intelligence”, “deep learning”, and “machine learning”, combined with “forensic medicine”, “legal medicine”, “forensic pathology” and, medicine”. We restricted our search to papers published in English and found more than 100 relevant manuscripts. The inclusion criteria focused on studies that examined the application of artificial intelligence in forensic medicine and pathology and various medical specialties.

### 2.2. Results

After a thorough review and assessment of the 113 articles, we identified and included a subset of 32 papers that were directly relevant to our research, including seven in forensic identification, one in Ballistics and additional factors of shooting, one in traumatic injuries, three in establishing the post-mortem interval, two in forensic toxicology, one in sexual assaults/rape, one in crime scene reconstruction, one in virtual autopsy, and fourteen in medical act quality evaluation, listed in Table 1. These selected studies provided valuable insights into the use and impact of AI in forensic medicine and pathology and various medical specialties, forming the basis of our review.

#### 2.2.1. Forensic Identification

Forensic odontostomatological identification by visual or clinical methods can sometimes be difficult. The expertise allows for the determination of ABO antigens, serum proteins (Gm, Km, Gc), enzyme markers, the genetic fingerprint, and the Y chromosome by analyzing the root pulp. Age and sex can be estimated by analyzing the dentin. Some authors have identified drugs, proteins, lipids, and carbohydrates in the tartar collected from corpses or from a living person, which can point to a certain population group through information related to socio-economic status, occupation, diet, dental, or systemic diseases [15,16]. However, the existing technique can be exhausting and complicated in a larger scale expertise, which requires a much larger number of forensic odontostomatological identifications. Mohammad N et al. state in the review carried out that the potential application of artificial intelligence in forensic odontology can be classified into four categories: (1) human bite marks, (2) sex estimate, (3) age estimation, and (4) dental comparison [1]. AI can provide a set of data, and a correctly assigned analysis algorithm in the case of hyperparameters, which allows for the creation of a predictive model at a very high level of performance.

**Table 1:** Scientific articles that analyze the use of artificial intelligence in forensic medicine.

Forensic Identification	**Year of Study**	**Author**	**Application**
	2022	Mohammad N. [17]	Forensic Odontology
	2020	Khanagar S.B. [18]	Forensic Odontology
	2021	Thurzo A. [19]	The AI impact on forensic anthropology
	2017	Nino-Sandoval T. [20]	Mandibular reconstruction
	2022	Matsuda S. [21]	Personal identification
	2016	Nguyen D.T [22]	Sex estimates
	2021	Massimo L. [23]	Visual semiotics and digital forensics
Ballistics and additional factors of shooting	2020	Bobbili R. [24]	Establishing the class of the weapon and the bullet caliber
Traumatic injuries	2005	Georgieva L. [25]	Estimating the ecchymosis age by color analysis
Post-mortem interval	2019	Hachem M. [26]	Prediction of PM interval through blood biomarkers
	2020	Zou Y. [27]	Post Mortem interval and AI
	2022	Wang Z. [28]	AI and microbiome for PM interval
Forensic toxicology	2020	Gasteiger J. [29]	Chemistry and AI
	2000	Helma C. [30]	Toxicology and AI
Sexual assaults/rape	2021	Golomingi R. [31]	Sperm identification under an optical microscope using AI
Crime Scene Reconstruction	2020	Siddhant G. [32]	Making animations regarding the circumstances of the death
Virtual autopsy	2017	O’Sullivan S. [33]; Gerke S. [34]	AI assistance in necropsy expertise
Medical Act Quality Evaluation	2019	Santin M. [35]	AI assistance in imaging investigations
	2020	Qui S. [36]	AI assistance in psychiatry
	2023 2019 2018	Salazar L. [37] Attia Z.I [38] Alsharqi M. [39]	AI assistance in cardiology
	2020	Zhang Y.H. [40]	Cancer management using AI
	2022	Rajpurkar P. [41]	AI assistance in pathology
	2020 2019	Young A.T [42] Dick V. [43]	AI in dermatology
	2020	Pedersen M. [44]	AI in neurology
	2017	Rathi S. [45]	AI in ophthalmology
	2021	Kroner P.T. [46]	AI in gastroenterology
	2015 2021	Idowu I.O. [47] Sone K. [48].	AI in obstetrics and gynecology

In Khanagar S.B. et al.’s review, we found the perspective of using AI for the reconstruction of the mandible [18]. These AI models are based either on artificial neural networks (ANN) or on convolutional neural networks (CNN). The results being promising, these models show accuracy and precision equivalent to those of experts in the field, useful especially in the case of the need to identify victims of mass disasters [17]. CNN is a deep learning algorithm that can apply different properties/aspects to an input image and differentiate it from others. Convolutional neural networks work like the human brain by trying to identify blurred images. Recognition improves when more slices are added, which provides a 3D feature [19,20]. A forensic anthropologist has the difficult task of storing and processing a huge amount of anthropometric information, and sometimes fatigue, subjectivism, methodological difficulties, and interpretation can lead to errors. The incidence of these errors can be reduced by processing data with the help of AI with automatic learning that imitates human neural networks which can solve even the most complex problems, without getting tired, without using emotions, and without particularizing cases through subjective judgment. During COVID-19, person identification (PI) with the help of AI would have been extremely useful, considering the number of deaths worldwide and the existence of unknown bodies that needed to be identified. Matsuda S. and Yoshimura H. carried out a scientific paper in 2022 in which they show that conventional anthropological methods that follow the description of facial features (hair, eye color, nose, lips, scars, tattoos, particular signs) or fingerprints and DNA analysis can be replaced with modern, new methods, with the involvement of artificial intelligence, already used for the identification of some bodily parameters, the retina model and fingerprints, especially in institutions where data security takes precedence, even proposing the use of artificial intelligence on all living people, ethically questionable methods, but which could help identify people [21]. Currently, out of all the identification methods used, facial recognition is in second place, after the fingerprint [22]. Although progress exists, and the replacement of fingerprint-based methods with AI identification devices requires training/learning with images of people, the human factor remains decisive in the formation of these systems, and the challenges related to the infrastructure and biometric resources of a population will exist [23]. 

#### 2.2.2. Ballistic Expertise and Additional Factors of the Shooting

When a bullet leaves a weapon, it carries microscopic traces, which are like ballistic fingerprints. Artificial intelligence can guide experts to the place where they need to look for gunpowder and cartridge tubes and compare the traces with a database through image processing, without human involvement. Currently, some algorithms allow for the highlighting the residues resulting from firing with firearms, allowing for the detection of the explosion inside the barrel, changes due to shock waves, as well as the provision of data that allow for the establishment of the class and caliber [24]. 

#### 2.2.3. Traumatic Injuries—Bruise Color’s Recognition

Ecchymoses undergo color changes in their evolution towards healing, through the degradation of hemoglobin: initially reddish, then violet-blue, on the third day it becomes greenish due to biliverdin, then after 4–5 days it turns brown due to bilirubin, and later turns yellow due to the accumulation of hemosiderin pigment, disappearing in 10–14 days. Forensic medicine assesses the age of the bruises initially macroscopically, passing the color change through this mind filter. The techniques for detecting bruise colors by AI are much more accurate than the human factor, which can be subjective in some cases, and through the created algorithm, a time interval of the production of traumatic injuries [25] is generated. 

#### 2.2.4. Determination of the Postmortem Interval 

Postmortem interval estimation (PMI) is part of the current, almost daily practice of forensics, being a very important expertise in some cases. An important step in the evolution of forensic investigations is the introduction of AI in PMI research. The field of biochemical technologies has begun to identify biomarkers in various biological fluids, such as blood and urine, for the estimation of PMI. It is suggested that the blood from the femoral vein should be collected for the measurement of biochemical components such as lactate dehydrogenase—LDH, aspartate aminotransferase—AST, triglycerides, and cholesterols, as well as the measurement of pH. These biochemical markers analyzed by artificial intelligence can provide information on the time of death [26]. After death, through the decomposition of the body, the level of these biomarkers changes and is directly proportional to the time elapsed since death. Zou Y et al. state that AI technology is in full development for data processing and is already being used by some researchers as a conventional method for PMI estimation [27]. By applying next-generation sequencing (NGS) and AI techniques, the forensic pathologist can enhance the dataset of microbial communities and obtain detailed information on the inventory of specific ecosystems, quantifications of community diversity, descriptions of their ecological function, and even their application in forensic medicine and pathology through post-mortem sequencing of the cadaveric microbiome [28].

#### 2.2.5. Forensic Toxicology

Artificial intelligence use prospects in forensic toxicology come from the idea of expanding the search field and creating links with millions of data to identify toxic substances, drugs, and different metabolites. Automated toxicological analysis through AI allowed for quantitative and qualitative identification, which by 2020 reached more than 160 million organic and inorganic substances found in the Chemical Abstract Service (CAS) database [29]. Helma C. et al. revealed in a scientific paper that there can be human errors by using the spectrophotometer, neutron, and high-performance liquid chromatography (HPLC), and in this sense, AI can play an essential role by providing a data set as a sample which will increase the precision of the method, the efficiency, and even the reduction in the costs of investigations [30].

#### 2.2.6. Sperm Identification

Sperm detection is an investigation that proves rape. In some cases, the samples contain no sperm or contain only small amounts of sperm. Therefore, the biological material is transferred to a glass slide and must be manually scanned using an optical microscope. This work can be very time-consuming, especially when no sperm are present. Convolutional neural networks trained by the VGG19 network and a variation of VGG19 with 1942 can fulfill this task, they can reduce the scanning time by locating the sperm on the microscope images [31].

#### 2.2.7. Crime Scene Reconstruction

The manual process of data collection, on-site research, and reconstruction often involves the forensic pathologist. Artificial intelligence can extract and analyze every aspect after it is given “some input data”, such as the corpse itself or any object next to it that could bring data on the death circumstances, by creating video animations [32].

#### 2.2.8. Virtual Autopsy

Artificial intelligence combined with virtual autopsy represents the latest trend in the field of forensic medicine and pathology. By teaching the machine to take images of the body through CT or MRI scans, the AI will identify the pathology of an organ, fractures, deep injuries, and types of inflammation and compare it to the database. The AI will also process these organic changes and form its own opinion regarding the anatomopathological diagnosis and the conclusions regarding the cause of death. This technique can also provide information on the injuries produced by firearms, measuring the entrance hole at the level of the flat bones, and comparing it with different models, thus allowing for the estimation of the caliber of the bullet. All this, together with the possibility of collecting biological samples through this method, can assist the forensic pathologist in issuing diagnoses and formulating more correct conclusions [33].

## 3. Discussion

### 3.1. Perspectives and Directions for the Application of Artificial Intelligence in Forensic Medicine and Pathology

The scientific palette in the field of artificial intelligence is still at the beginning and will certainly undergo changes to improve and assist the human factor in medicine. Forensic medicine is no exception to progress, and as we have shown through the review carried out, various methods are already being implemented worldwide, which we can subdivide into a few essential aspects: (1) assistance to the forensic pathologist regarding the accuracy of both the anatomopathological diagnosis macroscopically, as well as all complementary exams; (2) reducing subjective judgment and fatigue, all the factors that define human nature through its vulnerability; (3) reducing the costs that involve all the forensic activity by eliminating some investigations, sometimes necessary for the human with the help of AI that can express an earlier and more solid opinion, but also by eliminate the risk of repeating certain complementary examinations which can cause of human errors; (4) the contribution of artificial intelligence to the creation of an electronic data archiving environment, thus eliminating files and devices for storing and memorizing data, which are becoming more and more difficult to manage due to the volume they occupy and the fragility of USB drives and hard disk, as well as through the possibility of destroying these data. 

Although artificial intelligence is renovating the world, its legal value in front of a court is still not accepted. The human factor, although showing professional sensitivity, should have the last word to say in a forensic medical report. AI is still a technology in its teenage years, but it is already proving that its algorithms are more objective and smarter than humans, basically speculating on human weaknesses. From a scientific point of view, it is possible that the opinion formed by artificial intelligence will not be accepted as an individual conclusive proof, but probably with time and with the implementation of AI in our daily life, it will evolve and be unanimously accepted. Gerke S. et al. state that the challenges of artificial intelligence also include the ethical characteristics regarding the use of data, transparency and cyber security, algorithmic correctness, and the confidentiality of medical data [34].

### 3.2. Evaluation of Medical Malpractice Cases

The last topic we would like to touch on is the evaluation of medical malpractice with the help of artificial intelligence. Can we decrease the number of medical malpractice cases by using artificial intelligence in current practice? In the literature, we found numerous works that discuss the usefulness of AI in diagnosis accuracy, the prediction of some complications, as well as the establishment of the degree of effectiveness of the treatment, alongside works that outline this direction of research [35,36,37,38,39,40,41,42,43,44,45,46,47,48].

#### 3.2.1. Medical Diagnosis 

**Figure 1:**
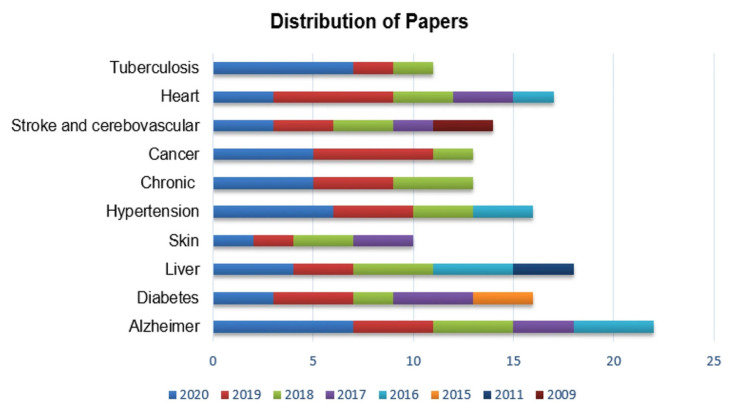
Distribution of published papers for disease diagnosis using artificial intelligence techniques [4]. Adapted from Kumar, Y., Koul, A., Singla, R. et al. Artificial intelligence in disease diagnosis: a systematic literature review, synthesizing framework, and future research agenda. J Ambient Intell Human Comput 14, 8459–8486 (2023).

AI can be trained to analyze medical images, such as X-rays, computed tomography (CT) scans, and magnetic resonance images (MRI), etc., (Figure 1) to detect and diagnose various pathologies [35,49,50,51,52].

#### 3.2.2. Personalized Treatment

Artificial intelligence and pharmacogenomics can help identify the right drugs for a patient based on their genes and potential drug–drug interactions; AI can analyze medical data, and history, to develop a predictive treatment plan [53].

#### 3.2.3. Disease Monitoring and Management

Monitoring systems can collect data about a patient’s condition (heart rate, glucose level, blood pressure, etc.) and artificial intelligence can analyze this data to detect early signs of complications. In a study conducted in China by Weng S.F. et al. between 2005 and 2015, using routine clinical data of over 350,000 patients, machine learning significantly improved the accuracy of cardiovascular risk prediction, correctly predicting 355 (an additional 7.6%) more patients who developed cardiovascular disease compared with the established algorithm [54]. 

In health data management, AI can help analyze and interpret massive amounts of healthcare data, providing healthcare professionals with relevant information and insights to make more informed decisions.

#### 3.2.4. Robot-Assisted Surgery

According to Zhou X.Y et al., artificial intelligence has revolutionized surgery, prolonging the life and survival limit of patients by managing acute and chronic diseases [55]. Current robots already can automatically perform some tasks related to basic surgery, such as suturing and knot tying [56,57]. In the literature, it has already described that a skilled surgical robot was able to completely suture the small intestines of a pig on its own, surpassing the human manual dexterity of experienced surgeons who performed the same suture at the same time [58].

#### 3.2.5. Drug Discovery

With all the current progress, artificial intelligence could accelerate the process of new drug discovery, as its ability to model molecular interactions and identify compounds with potential therapeutic properties is already known. For example, in toxicology, deep learning might automatically identify high-level drug use patterns by combining data from social media, poison control logs, published reports, and national surveys [59].

All that remains is to deepen this perspective and the limits of AI, in the hope that it will provide us with useful data comparable to a complementary forensic medical examination, helping human nature to reduce its errors, all of which will be reflected in the benefit of patients and the whole society.

## 4. Conclusions

Advancements in artificial intelligence (AI) have marked a turning point in the field of forensic medicine and pathology. In recent years, these technologies have demonstrated significant potential in optimizing the processes of data analysis and interpretation. However, it is important to emphasize that human expertise remains essential in making critical decisions. One of the major challenges in using AI in this context is ensuring the quality of scientific data. The process of collecting and cleaning data is of fundamental importance to ensure the performance and accuracy of AI algorithms. Only by providing reliable data can we reach the maximum potential of this technology in forensic medicine and pathology.

The benefits brought by AI within the justice system are evident. These include accelerating the analysis of evidence and interpreting clues, which can lead to a more efficient and precise legal process. However, it is crucial to recognize that the transition to fully automated AI use will take time and continuous adjustments. Fundamentally, AI is not a substitute for human expertise, but rather a valuable partner. Human discernment and knowledge remain indispensable for interpreting context and making informed decisions. By balancing technology with human expertise, we can maintain a fair and ethical justice system in an ever-evolving modern world.

Thus, the progressive integration of AI in forensic medicine and pathology represents a significant step towards improved well-being and security for society. Through the collaboration between technology and human expertise, we can fully harness the advantages offered by this innovation in the fields of justice and forensic medicine.

## Data Availability

Not applicable.
